# Sonic Hedgehog Signaling Pathway Supports Cancer Cell Growth during Cancer Radiotherapy

**DOI:** 10.1371/journal.pone.0065032

**Published:** 2013-06-10

**Authors:** Jingjing Ma, Ling Tian, Jin Cheng, Zhiwei Chen, Bing Xu, Liwei Wang, Chuanyuan Li, Qian Huang

**Affiliations:** 1 Experimental Research Center, First People’s Hospital, School of Medicine, Shanghai Jiaotong University, Shanghai, China; 2 Department of Radiation Oncology, First People’s Hospital, School of Medicine, Shanghai Jiaotong University, Shanghai, China; 3 Department of Oncology and Laboratory for Pancreatic Diseases, First People’s Hospital, School of Medicine, Shanghai Jiaotong University, Shanghai, China; 4 The Department of Dermatology, Duke University Medical Center, Durham, North Carolina, United States of America; Indiana University School of Medicine, United States of America

## Abstract

Tumor growth after radiotherapy is a commonly recognized cause of therapeutic failure. In this way, we examined tumor cell growth after radiotherapy by establishing a cancer cell growth model *in vitro*. We accomplished this model by seeding non-irradiated firefly luciferase2 and green fluorescent protein fusion gene (Fluc) labeled living cancer reporter cells onto a feeder layer of irradiated cancer cells. The living tumor cell growth was monitored by bioluminescence imaging. The living reporter cells grew faster when seeded onto lethally irradiated feeder cells than when seeded onto non-irradiated feeder cells or when seeded in the absence of feeder cells. We found that the expression levels of the Shh and Gli1 proteins, both of which are critical proteins in Sonic hedgehog (SHH) signaling, were increased after irradiation and that this expression was positively correlated with reporter cell growth. Moreover, the dying cell stimulation of living tumor cell growth was enhanced by the addition of SHH signaling agonists and inhibited by SHH signaling antagonists. SHH agonists also enhanced reporter cell growth in the absence of irradiated feeder cells, suggesting this mechanism plays a role in feeder cell growth stimulation. Given these results, we conclude that SHH signaling activation plays an important role during tumor repopulation after radiotherapy.

## Introduction

In many types of cancer, tumor repopulation after radiotherapy occurs and poses a major challenge for clinicians. In this process, the few tumor cells that survive after radiotherapy will regrow and replace lost tumor cells. Repopulation during fractionated radiotherapy is recognized as an important cause of treatment failure and evidence suggests that the rate of repopulation may occur at an accelerated pace in some cases [1]. Repopulation depends on the activation of signaling pathways that stimulate the proliferation of tumor cells and many molecular-targeted agents have been developed that inhibit these pathways, such as gefitinib which targets epidermal growth factor receptor (EGFR) signaling [2]. In order to create a model for studying tumor cell repopulation after radiotherapy, many others have tried using so-called “feeder cells” that have been treated with radiation to promote the growth of untreated tumor cells seeded in co-culture experiments. Although this model does not directly show repopulation of treated cancer cells during radiotherapy, we believe that the growth-promoting signals released from lethally treated feeder cells replicate the conditions in a homogenously treated tumor. Therefore, we will use the concept of repopulation and dying cell stimulated living tumor cell growth synonymously throughout our study.

Despite our knowledge that dying feeder cells may increase the growth of living tumor cells, the mechanism by which this occurs remains largely unknown. The sonic hedgehog (SHH) signaling pathway, first identified in Drosophila melanogaster, is implicated in normal organ development and homeostasis, stem cell maintenance and proliferation in vertebrates and may therefore be a candidate for the mechanism behind surviving tumor cell growth [3]. Moreover, many cancers are associated with SHH signaling, such as basal-cell carcinoma, esophageal and stomach cancer, small-cell lung cancer [4] and pancreatic adenocarcinoma [5]. SHH pathway activation is initiated by binding of the secreted and lipid-modified ligand Shh to Patched1 (Ptch1) transmembrane receptor. As a consequence, Ptch1 inhibition of Smoothened (Smo), a 7-pass transmembrane-spanning protein, is relieved and the SHH signaling cascade is initiated, which in turn activates the Gli transcription factors [6]. There are three Gli proteins that encode both activator and repressor function. Gli1 acts as a transcriptional activator, Gli2 is a composite of positive and negative regulatory domains, and Gli3 acts primarily as a transcriptional repressor [7]. In the presence of Shh, Gli1 is transcriptionally activated and the phosphorylated and proteolytical processing of Gli2 and Gli3 to their truncated repressor forms is inhibited, thus leading to the activation of specific SHH signaling pathway target genes, such as Gli1 and Ptch1 [8].

Since the mechanisms underlying tumor accelerated repopulation during radiotherapy are not well understood, we aim to investigate a role for the well-established SHH pathway in the tumor cell proliferation after radiotherapy process. It is well known that radiotherapy causes apoptosis which may play a critical role in tumor cell repopulation [9]. In our previous studies, we have shown that dying tumor cells use the apoptotic process to generate caspase 3 mediated growth-stimulating signals to stimulate the repopulation of tumors undergoing radiotherapy [10]. Furthermore, we also found “Phoenix Rising” pathway through which executioner caspases, such as Caspase 3 and 7, in apoptotic cells promote wound healing and tissue regeneration in multicellular organisms [11]. In esophageal cancer, the SHH signaling pathway was extensively activated in xenografts and residual tumors after chemoradiotherapy and blocking SHH signaling enhanced radiation cytotoxicity [12]. Therefore, the “Phoenix Rising” pathway with caspase-mediated tumor growth stimulation and the SHH signaling pathway may both be involved in tumor cell repopulation after radiotherapy.

In this study, we examined the roles of SHH signaling pathway in dying cell stimulated tumor cell growth. Our data shows clear evidence for a role for Shh secreted by dying cells in promoting the rapid repopulation of tumors from a small number of living tumor cells. We believe this newly discovered pathway of Shh stimulated tumor repopulation plays a key role in cancer radiotherapy. Furthermore, targeting the SHH pathway may have clinical implications for the improvement of cancer radiotherapy outcomes.

## Materials and Methods

### Cell Culture Conditions

Human pancreatic cancer Panc1 cells and human colonic cancer HT29 cells, were purchased from the Chinese Academy of Science (Shanghai, China) and cultured in Dulbecco’s Modified Eagles’s Medium (DMEM) (Thermo, Beijing, China) with 10% fetal bovine serum (FBS, Sijiqing, Hangzhou, China), 100 U ml^–1^ penicillin, and 100 µg ml^–1^ streptomycin at 37°C in humidified atmosphere containing 5% CO_2_.

### Tumor Repopulation Model

*In vitro*, HT29 cells or Panc1 cells cultured in 10 cm petri dish were X-ray irradiated and twenty-four hours later, they were trypsinized and seeded into 24 well plates (Corning, NewYork, USA) at a density of 2.5×10^5^ cells per well in triplicate in DMEM containing 2% FBS. Twenty-four hours later, Fluc labeled, untreated HT29 or Panc1 cells were seeded at a cell density of 1000 cells per well. The medium was changed every 2 days for 14 days.

### Irradiation of Cancer Cells

X-ray irradiation of cells was carried out using an Oncor linear accelerator (Siemens, Amberg, Germany), located in the department of radiation oncology at Shanghai Jiaotong University affiliated First People’s Hospital. The dose rate for the machine is about 3.6 Gy/min.

### Gene Transduction into the Cells

We transduced various exogenous genes into target cells by use of lentivirus vectors. The most commonly used lentiviral vector is the pLEX system (Thermo Scientific Inc, Beijing, China), which contained a puromycin resistance gene. Genes that were cloned into this vector include the following: firefly luciferase2 and green fluorescent protein fusion gene (Fluc) driven by CMV promoter, luciferase gene driven by 8× wild-type Gli1 binding site or 8× mutated Gli1 binding site (i.e. wild-type 8× GBS luciferase gene or mutated 8× GBS luciferase gene), as well as shRNA against Gli1. All the lentiviral vectors were packaged in 293T cells following manufacturer’s instructions. The stably transduced HT29 or Panc1 cells were obtained by lentivirus infection and puromycin selection in the presence of 2 µg/ml puromycin for two weeks.

### Luciferase Assay

The Luciferase Reporter Assay System E1500 (Promega, Wisconsin, USA) was used to determine firefly luciferase activities according to the manufacturer’s instructions. HT29 and Panc1 cells expressing the wild-type 8× GBS luciferase gene or the mutated 8× GBS luciferase gene were irradiated with 6 Gy of ionizing radiation. The luciferase activities for the 6 Gy irradiated cells and non-irradiated cells were tested. Measurements were performed with a Berthold luminometer (Berthold Technologies, Bad Wildbad, Germany), and firefly luciferase values of cells expressing wild-type luciferase gene were normalized by firefly luciferase values of cells expressing mutant luciferase gene. Normalization minimizes experimental variability. All experiments were performed in triplicate and then repeated three times.

### Bioluminescence Imaging

To image the luciferase signals emitted from cells, we used the NC100 instrument from Berthold Technologies (Bad Wildbad, Germany) located in School of Basic Medical Sciences, Fudan University. For Panc1 and HT29 cells, we measured luciferase signals by adding D-luciferin (Promega, Wisconsin, USA) in PBS at a final concentration of 0.15 mg/ml. Five minutes after the administration of D-luciferin, the images were taken and luciferase signals (photons/sec) were then processed and analyzed quantitatively by use of manufacturer supplied software. Images were always taken at the same time point to minimize variability. The luciferase signals were measured in Panc1 and HT29 cells at 14 day time point to maximize the observed difference between the no feeder and untreated controls from the irradiated feeder experimental group.

### Antibodies and Key Chemicals Used in this Study

Commercially available antibodies against Shh, Gli1, β-actin, GAPDH (Cell Signaling Technology, Boston, USA) and secondary antibody conjugated with horseradish peroxidase (Bio-Rad, California, USA) were purchased. SHH signaling antagonist cyclopamine and Gant61 were both obtained from Sigma-Aldrich (Missouri, USA). GDC-0449 was purchased from Selleck Chemicals (Texas, USA). SHH signaling agonist SAG was obtained from Enzo Life Sciences (NewYork, USA) and recombinant mouse sonic hedgehog N-terminus was obtained from R&D Systems (Minnesota, USA).

### Agonist and Antagonist Treatment

Agonists or antagonists were added when irradiated HT29 or Panc1 cells were seeded into 24 well plates as feeders. The following concentrations were used: cyclopamine for HT29 cells (2 µM and 5 µM), cyclopamine for Panc1 cells (0.5 µM, 1 µM, 2 µM and 5 µM), GDC-0449 for HT29 cells (0.2 µM, 0.5 µM, 1 µM and 2 µM), GDC-0449 for Panc1 cells (0.5 µM, 1 µM and 2 µM), Gant61 for HT29 cells (1 µM, 2 µM and 5 µM), Gant61 for Panc1 cells (2 µM, 5 µM, 10 µM and 20 µM), recombinant mouse sonic hedgehog N-terminus for HT29 and Panc1 cells (600 ng/ml), SAG for HT29 cells (5 nM, 10 nM and 100 nM), SAG for Panc1 cells (3 nM, 5 nM, 10 nM and 100 nM). To confirm the effect of agonists on live tumor cells, agonists were also added into wells containing HT29 or Panc1 reporter cells alone. The concentrations were used as: SAG for Panc1 and HT29 cells (5 nM, 10 nM and 100 nM), recombinant mouse sonic hedgehog N-terminus peptide for HT29 and Panc1 cells (600 ng/ml). Medium was changed every forty-eight hours and replaced with fresh medium containing same concentration of agonists or antagonists. The growth of reporter cells was monitored 14 days later by imaging.

### ShRNA Knockdown

The lentiviral plasmids encoding shRNA against Gli1 gene (HSH007701-HIVmU6) and scramble control (CSHCTR001-HIVmU6) were purchased from Genecopoeia (Maryland, USA). The sequence for shRNA against Gli1 is 5′-acgccatgttcaactcgat-3′. Lentiviruses encoding Gli1 shRNA and scramble control were produced as described above. HT29 and Panc1 cells were seeded in six well plates and subsequently infected. The cells were then treated with puromycin to select for those stably expressing shRNA against Gli1 and scramble control RNA. Silencing efficiency was confirmed using Western blot for Gli1 protein.

### Western Blotting

Cells were washed twice with PBS and lysed using 120–200 µl standard RIPA buffer containing protein inhibitors (Beyotime, Jiangsu, China). Protein concentration of each sample was quantified and 40–60 µg protein per sample was used for Western blot analysis. In general, 40–60 µg protein in loading buffer was heated to 100°C for 10 minutes and then separated in a SDS-polyacrylamide gel by electrophoresis, and transferred to a PVDF membrane (Bio-Rad, California, USA). The membranes were incubated with primary antibodies overnight at 4°C and then with secondary antibodies for 2 hours at room temperature. ECL Plus (Roche, Basel, Switzerland) was used to visualize the signals on the membrane.

### Statistical Analysis

Results were analyzed using one-way analysis of variance (ANOVA) test to assess statistical significance respectively, with values of *P<*0.05 considered statistically significant. All statistical analyses were performed with SPSS 13.0 (SPSS Inc., Chicago, IL).

## Results

### Reporter Cell Numbers were Linearly Associated with Luciferase Activity When Imaging

**Figure 1:**
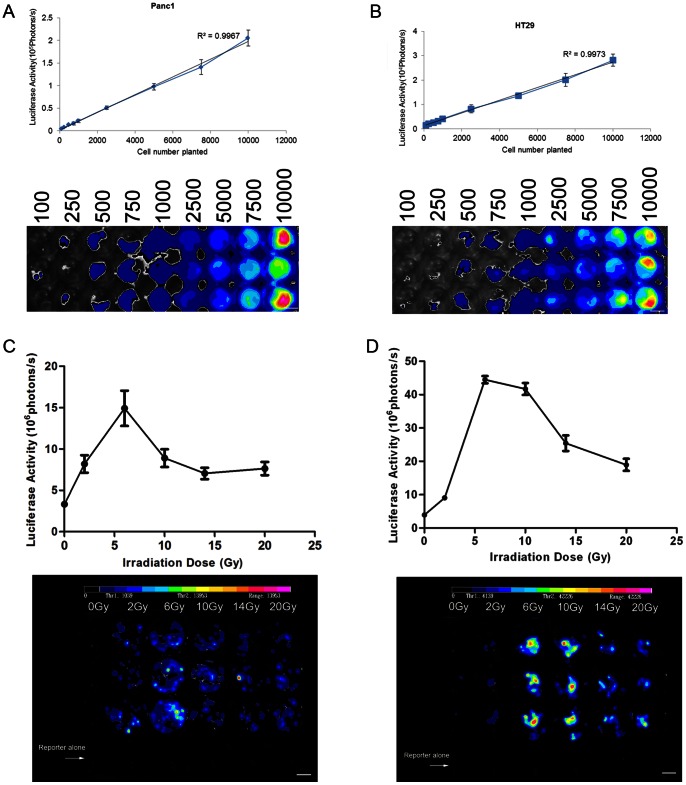
Growth-Stimulating properties of dying tumor cells irradiated at various doses. A. Correlation analysis of photons measured by bioluminescence imaging *vs* cell numbers plated. Fluc labeled Panc1 cells were plated in 96 well plates in 6 repeats at the number indicated the day before Imaging. The average photons/sec from 6 repeats at each cell numbers were analyzed by two-tailed ANOVA (R^2^ = 0.9967). The results indicated that intensity of image signal positively correlated with cell numbers plated. White scale bar represents 1 cm in length. B. Correlation analysis of photons measured by bioluminescence imaging *vs* cell numbers in HT29 cells. The procedure and result analysis were as same as Panc1 cells mentioned above (R^2^ = 0.9973). Scale bar represents 1 cm. C. Analysis of signal intensity of Panc1Fluc cells grown on irradiated Panc1 cells. 2.5×10^5^ X-ray irradiated Panc1 cells were plated into 24 well plates as feeder. The doses were 0 Gy, 2 Gy, 6 Gy, 10 Gy, 14 Gy and 20 Gy respectively. 1000 Panc1Fluc cells were plated into each well with or without feeder cells as reporter. 14 days later plate was imaged for bioluminescence intensity. Top: Luciferase activity analysis; Bottom: representative bioluminescence image, scale bar represents 1 cm. D. Analysis of signal intensity of HT29Fluc cells grown on irradiated HT29 cells. The procedure and result analysis were as same as Panc1 cells mentioned above. Top: Luciferase activity; Bottom: representative bioluminescence image, scale bar represents 1 cm.

In order to confirm the correlation of luciferase activity in images with reporter cell numbers, we did a series of dilution for Fluc labeled tumor cells (termed “reporter cells”). 100, 250, 500, 750, 1000, 2500, 5000, 7500 and 10000 Panc1Fluc or HT29Fluc tumor cells were seed into 96 well plates in 6 replicates the day before imaging. The imaging was performed 5 minutes after adding D-luciferin using the NC100 instrument. The photons from each well were collected and subsequently analyzed by two-tailed ANOVA. The results indicated that photons/sec were linearly associated with cell numbers seeded in wells (Fig. 1A, 1B, R ^2^ = 0.9967 in Panc1Fluc cells and R^2^ = 0.9973 in HT29Fluc cells respectively).

### Irradiated Dying Tumor Cell Stimulated Living Tumor Cell Growth

We carried out a series of experiments to examine the effects of dying, irradiated tumor cells at various doses on living tumor cells. To simulate *in vivo* scenarios where the vast majority of tumor cells are killed by radiation or chemotherapy, we seeded a small number (10^3^) of Fluc labeled human pancreatic cancer Panc1 cells or human colonic cancer HT29 cells onto a bed of a much larger number (2.5×10^5^) of unlabeled homologus cancer cells. The latter cancer cells termed “feeder cells” were irradiated at 2 Gy, 6 Gy, 10 Gy, 14 Gy and 20 Gy, or untreated (0 Gy) respectively. Growth of the small number of living “reporter cells” was monitored by epi-fluorescent microscopy at 3 day intervals and by bioluminescence imaging on day14 (Fig. 1C, 1D). Luciferase activities were used as surrogates for the number of “reporter cells” which was verified by our linear association experiment (Fig. 1A, 1B). Our results indicated that reporter cells grew significantly faster when seeded onto dying cells than when seeded alone. In addition, feeder cells irradiated with 6 Gy showed the highest growth enhancing ability than other doses did, with non-irradiated feeder cells showing no supportive role. In tumor cells irradiated with doses higher than 6 Gy, growth stimulating ability was reduced with increasing irradiation dose (Fig. 1C, 1D). These observations were true for both HT29 cells and Panc1 cells.

### Activation of SHH Signaling Pathway Correlated Positively with Dying Cell Stimulated Living Tumor Cell Growth

**Figure 2:**
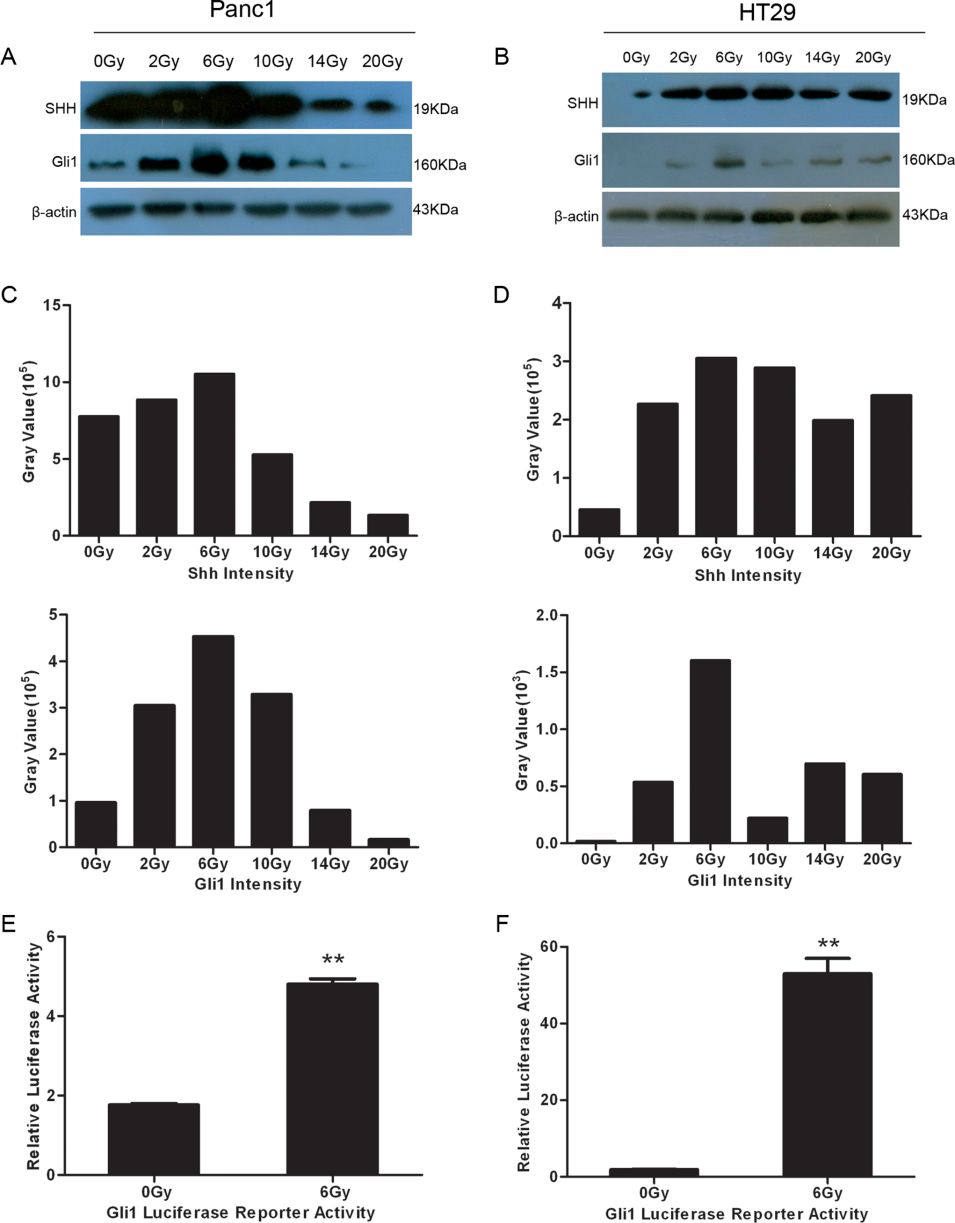
Evidence for SHH signaling pathway activation in irradiated Panc1 and HT29 cells. A. Expression-profile changes of Shh and Gli1 proteins in Panc1 cells irradiated at various doses and detected by Western blot. B. Expression-profile changes of Shh and Gli1 proteins in HT29 cells irradiated at various doses and detected by Western blot. C. Relative intensity of Shh and Gli1 protein bands on Western blot in Panc1 cells irradiated at various doses. D. Relative intensity of Shh and Gli1 protein bands on Western blot in HT29 cells irradiated at various doses. E. Luciferase activity of Gli1 reporter in irradiated and non-irradiated Panc1 cells. **represents *P*<0.01. F. Luciferase activity of Gli1 reporter in irradiated and non-irradiated HT29 cells. **represents *P*<0.01.

To examine whether SHH signaling pathway activation was associated with stimulation of tumor cell growth by dying cells, we carried out Western blot experiments with two cancer cell lines, Panc1 (Fig. 2A) and HT29 (Fig. 2B). Activated SHH signaling was confirmed by the protein levels of Shh and Gli1 which were quantified by measuring the signal of the 19-kD and 160-kD bands, respectively. We found that the levels of Shh and Gli1 proteins were higher in 6 Gy irradiated cancer cells than other doses treated cancer cells (Fig. 2C, 2D). Furthermore, in tumor cells irradiated with doses higher than 6 Gy, Shh and Gli1 protein levels were reduced with the increment of irradiation dose. It is interesting that the trends in protein expression level of the SHH signaling pathway exhibited the same tendency with the growth stimulation effect after irradiation, both of which were highest for 6 Gy and tapered off with increasing irradiation dose.

To further confirm the activation of SHH signaling pathway in the feeder cells, Panc1 and HT29 cancer cells were transduced with lentivirus carrying a wild-type 8× GBS luciferase reporter or a mutated 8× GBS luciferase reporter harboring a point mutation that abolishes the binding of Gli1. The cells infected by lentivirus were selected with 2 µg/ml puromycin. The stably transduced Panc1 and HT29 cells were untreated or irradiated at a dose of 6 Gy, and then luciferase activity was measured. The results suggested that the relative luciferase activity in 6 Gy irradiated cancer cells was significantly higher than that in non-irradiated cancer cells (*P<*0.01), indicating that Gli1 transcriptional factor activity was increased in 6 Gy irradiated cancer cells. The results that were observed in both Panc1 cells (Fig. 2E) and HT29 cells (Fig. 2F) were similar and consistent with results from bioluminence imaging shown above.

### SHH Signaling Antagonists Inhibit Dying Tumor Cell Stimulated Living Tumor Cell Growth

**Figure 3:**
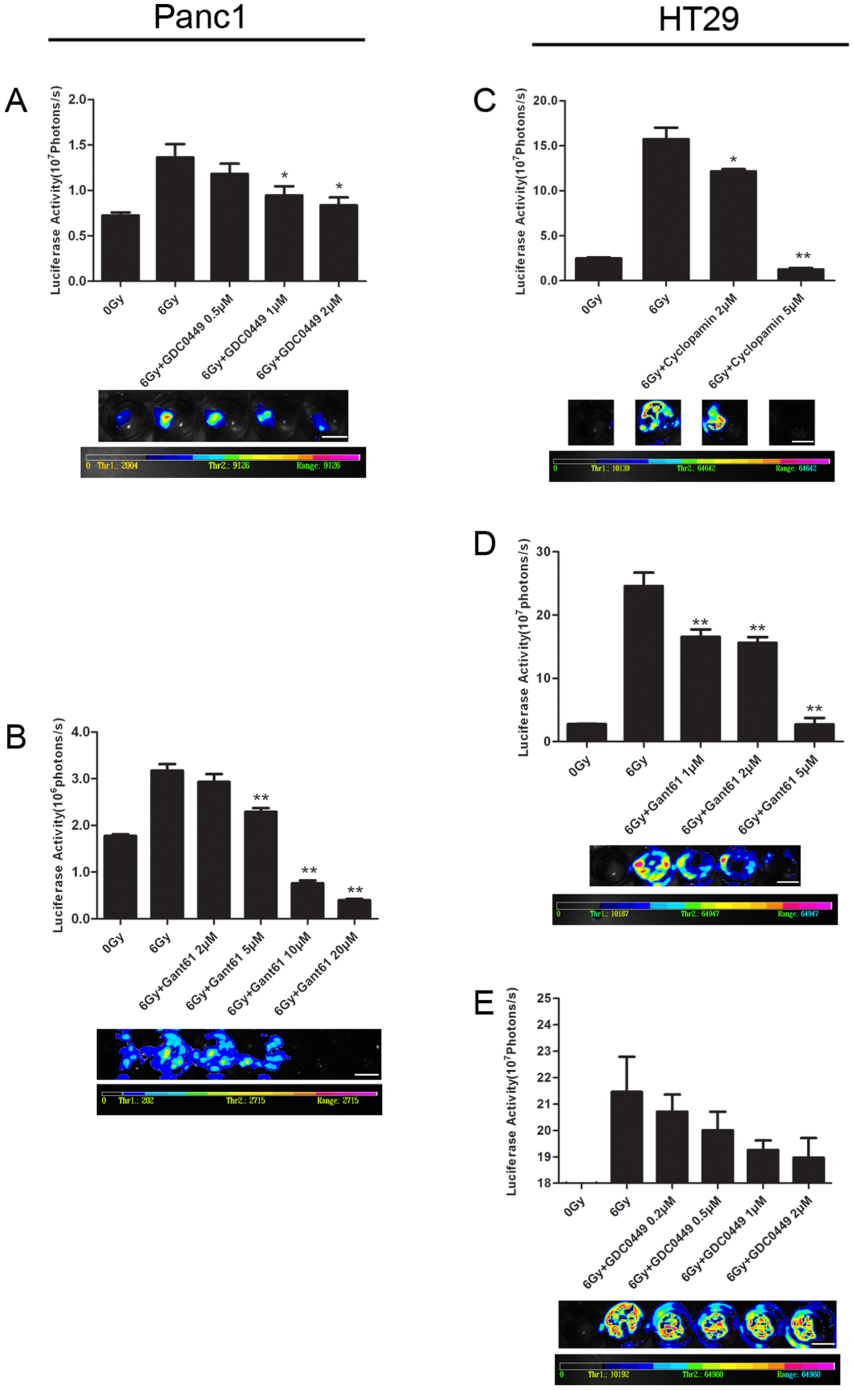
Effects of SHH signaling antagonists on dying cell induced tumor cell repopulation. A. GDC0449, a preclinical SHH pathway inhibitor, inhibits Panc1Fluc cell growth induced by dying Panc1 cell in a dose-dependent manner. Top: signal intensity analysis from bioluminescence image; Bottom: representative bioluminescence images; scale bar represents 1 cm. *represents *P*<0.05. B. Gant61 inhibits Panc1Fluc cells growth induced by dying Panc1 cells in a dose-dependent manner. Top: signal intensity analysis from bioluminescence image; Bottom: representative bioluminescence images; scale bar represents 1 cm. **represents *P*<0.01. C. Cyclopamin inhibits HT29Fluc cell growth induced by dying HT29 cells in a dose-dependent manner. Top: signal intensity analysis from bioluminescence image; Bottom: representative bioluminescence images; scale bar represents 1 cm. *represents *P*<0.05, **represents *P*<0.01. D. Gant61 inhibits HT29Fluc cell growth induced by dying HT29 cells in a dose-dependent manner. Top: signal intensity analysis from bioluminescence image; Bottom: representative bioluminescence images; scale bar represents 1 cm. **represents *P*<0.01. E. GDC0449 inhibits HT29 Fluc cell growth induced by dying HT29 cells in a dose-dependent manner. Top: signal intensity analysis from bioluminescence image; Bottom: representative bioluminescence images; scale bar represents 1 cm.

Given the significantly up-regulated SHH pathway activity in irradiated cells, we examined whether manipulation of the SHH pathway would inhibit or promote dying tumor cell stimulated living tumor cell growth. About 2.5×10^5^ 6 Gy irradiated Panc1 cells were seeded into 24 well plates in medium containing Smo antagonist (GDC-0449) at 0.5 µM, 1 µM, 2 µM or vehicle control respectively. 1000 Fluc labeled live Panc1 cells were seeded onto the irradiated with or without drug treated feeder layer. As shown in Fig. 3A GDC-0449 reduced Panc1 cells growth in a dose-dependent manner. Compared with controls which contained vehicle, the signal in wells which contained 1 µM GDC-0449 or 2 µM GDC-0449 was significantly reduced (*P*<0.05). These findings suggest that GDC-0449 could inhibit dying tumor cell stimulated living tumor cell growth.

To further confirm the roles of SHH signaling on dying tumor cell stimulated living tumor cell growth, we tested another Gli1 antagonist (Gant61). The condition was identical to the aforementioned condition for GDC-0449 except that the Gant61 concentration was either 5 µM, 10 µM or 20 µM. We observed a similar reduced growth in Gant61 treated wells compared with vehicle treated control wells (*P*<0.01, Fig. 3B). However, Panc1 cells treated with another Smo antagonist (cyclopamine) did not show a reduction in cell growth (data not shown).

Similar experiments were conducted in HT29 cells. About 2.5×10^5^ 6 Gy irradiated HT29 cells were seeded into 24 well plates in medium with or without cyclopamine at 2 µM, 5 µM or vehicle control respectively, onto which 1000 Fluc labeled live HT29 cells were seeded. Compared with vehicle control treated group, cyclopamine reduced HT29 cell growth in a dose-dependent manner (Fig. 3C). The HT29 cells grown in vehicle control showed a significantly greater luciferase activity than those cells grown in 2 µM cyclopamine (*P<*0.05) and 5 µM cyclopamine (*P*<0.01).

We further tested the Gli1 antagonist Gant61 (Fig. 3D) and the Smo antagonist GDC-0449 (Fig. 3E). In both cases, similar results were observed. The Gli1 antagonist Gant61 inhibited HT29 growth on dying feeder cells significantly. However, the difference between the vehicle control group and the GDC-0449 treated groups was not significant (*P*>0.05).

### Gli1 Knockdown by shRNA Reduces Dying Tumor Cell Stimulated Living Tumor Cell Growth

**Figure 4:**
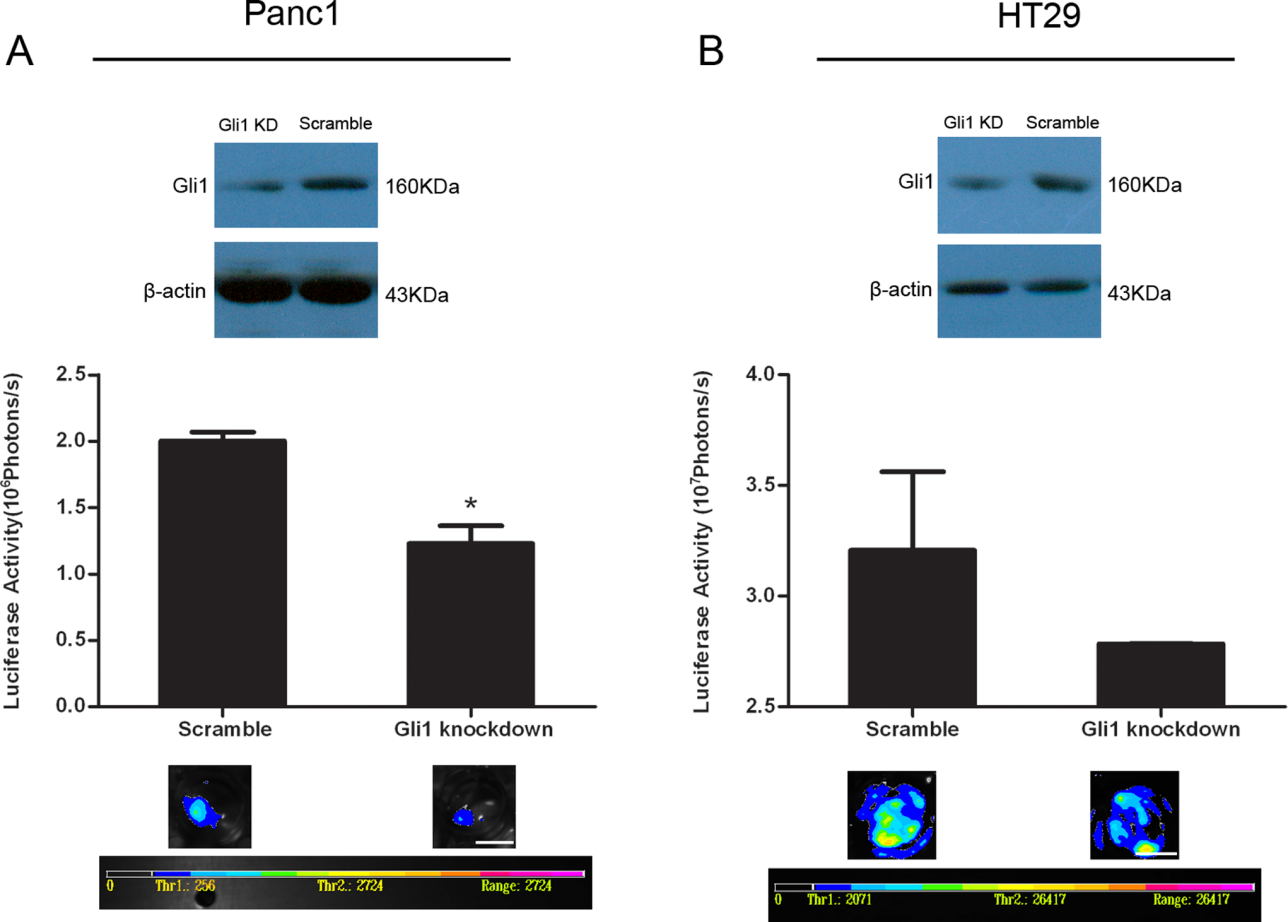
Effects of Gli1 knockdown on dying cell induced tumor cell growth. A. The reduced Gli1 protein expression in Gli1 shRNA transduced Panc1 cells detected by Western blot (upper panel). The reduced Panc1Fluc cell growth on dying Gli1 shRNA transduced Panc1 cells. *represents *P*<0.05; scale bar represents 1 cm. B. The reduced Gli1 protein expression in Gli1 shRNA transduced HT29 cells detected by Western blot (upper panel). The reduced HT29Fluc cell growth on dying Gli1 shRNA transduced HT29 cells. scale bar represents 1 cm.

We further confirmed the role of SHH signaling in dying tumor cell stimulated living tumor cell growth by using shRNA to knockdown Gli1 expression in feeder cells. HT29 and Panc1 cells infected with lentivirus carrying Gli1 shRNA were selected in media with 2 µg/ml puromycin, and Western blot analysis for Gli1 expression was used to verify proper selection. The protein levels of Gli1 in both Panc1 and HT29 cells (Fig. 4A, 4B) were markedly reduced. Panc1 or HT29 cells transduced by Gli1 shRNA or scramble shRNA were irradiated with 6 Gy and used as feeder cells, respectively. The growth of Fluc labeled living Panc1 or HT29 cells seeded onto Gli1 knockdown feeder cells was significantly attenuated as evidenced by the significantly lower luciferase activities in wells with Gli1 knockdown Panc1 or HT29 cells than wells with scramble shRNA transduced Panc1 or HT29 cells (*P*<0.05) (Fig. 4A, 4B).

### SHH Signaling Agonists Activate Tumor Cell Growth

**Figure 5:**
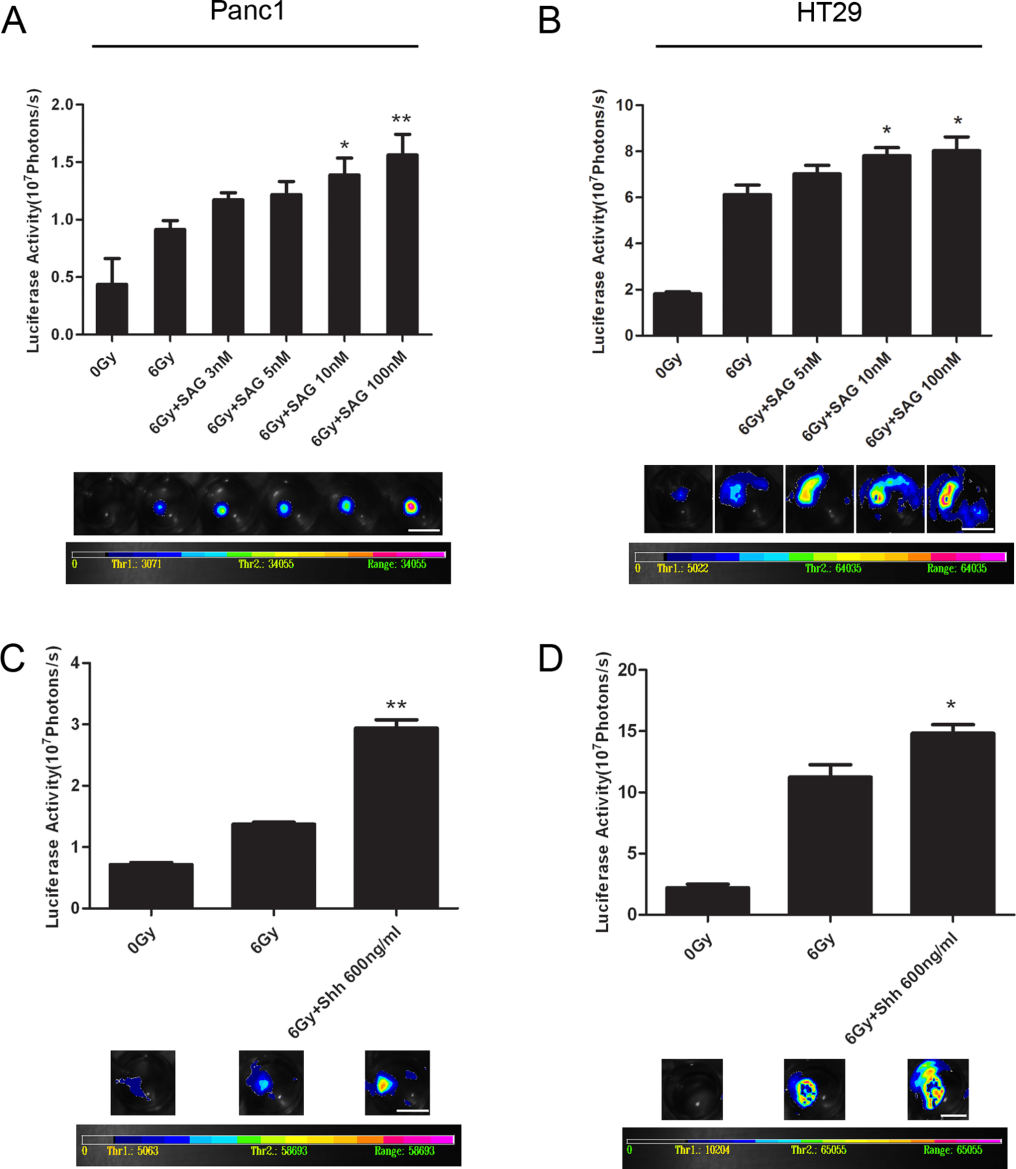
Effects of SHH signaling agonists on dying cell induced tumor cell growth. A. Activation of SHH signaling with SHH signaling agonist SAG enhances Panc1Fluc cell growth on irradiated dying Panc1 cells in a dose-dependent manner. Top: signal intensity analysis from bioluminescence image; Bottom: representative bioluminescence images; scale bar represents 1 cm. *represents *P*<0.05, **represents *P<*0.01. B. Activation of SHH signaling with SAG enhances HT29Fluc cell growth on irradiated dying HT29 cells in a dose-dependent manner. Top: signal intensity analysis from bioluminescence image; Bottom: representative bioluminescence images; scale bar represents 1 cm. **represents *P<*0.01. C. Activation of SHH signaling with SHH signaling agonists, N-terminal fragment of Shh, enhances Panc1Fluc cell growth on irradiated dying Panc1 cells. Top: signal intensity analysis from bioluminescence image; Bottom: representative bioluminescence images; scale bar represents 1 cm. *represents *P*<0.05. D. Activation of SHH signaling with N-terminal fragment of Shh enhances HT29Fluc cell growth on irradiated dying HT29 cells. Top: signal intensity analysis from bioluminescence image; Bottom: representative bioluminescence images; scale bar represents 1 cm. *represents *P*<0.05.

We next inquired if the SHH signaling pathway agonist, SAG, which acts by directly binding to downstream Smoothened, would promote cancer cell growth. Panc1 and HT29 cells were irradiated at 6 Gy and seeded in 24 well plates as feeders in medium with or without 3 nM, 5 nM, 10 nM or 100 nM SAG respectively. SAG treatment resulted in increased reporter cell growth in a dose-dependent manner (Fig. 5A, 5B). To further verify the results obtained with SAG, we added an active form of Shh, i.e. recombinant N-terminal fragments of Shh at 600 ng/ml into the medium. The results suggested that the recombinant N-terminal fragment of Shh significantly enhanced tumor cell growth on dying feeder cells in comparison with vehicle control (*P*<0.01) (Fig. 5C, 5D).

### SHH Signaling Agonists Enhance Living Reporter Cell Growth in the Absence of Irradiated Feeder Cells

**Figure 6:**
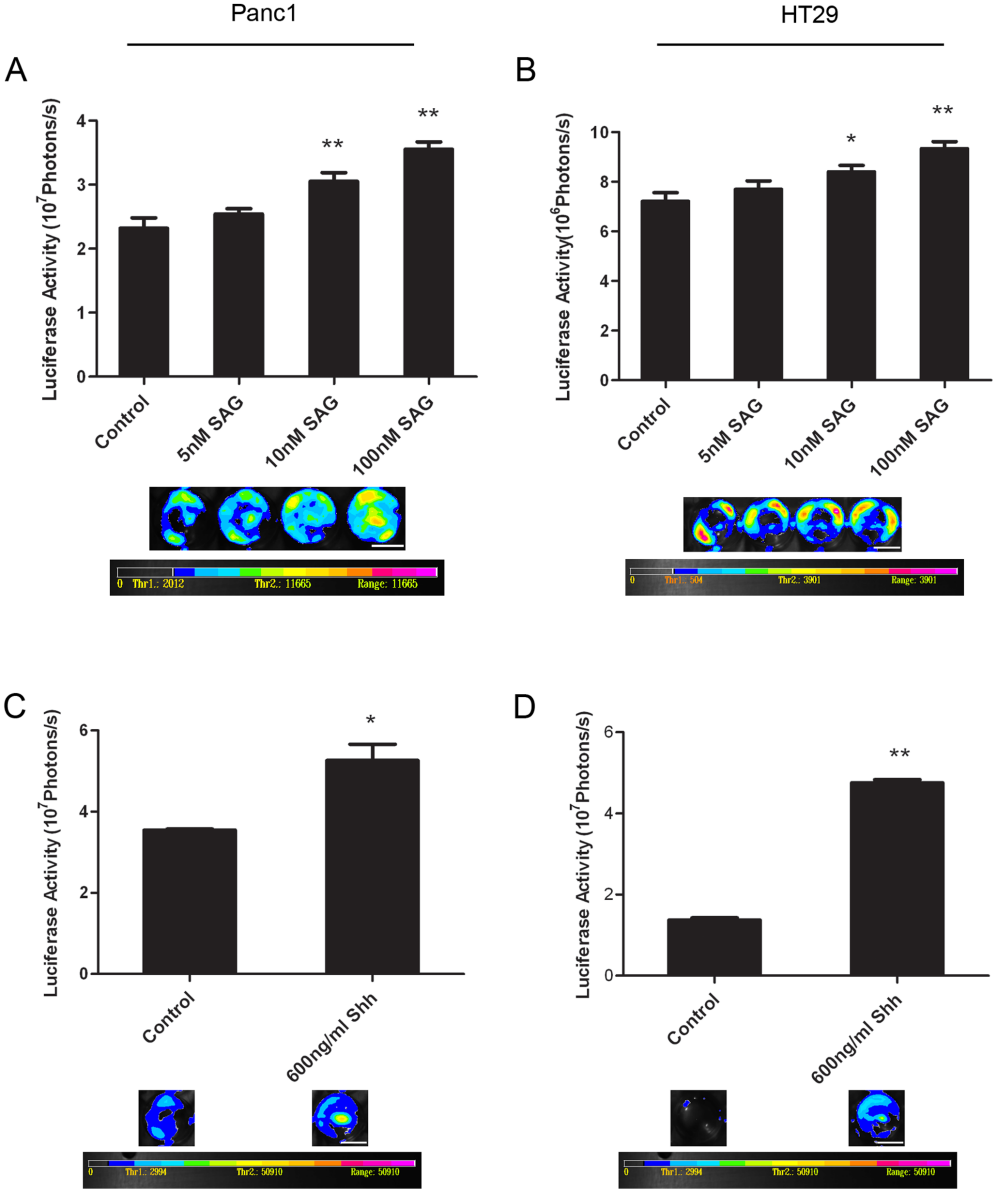
Effects of SHH signaling agonists on tumor cell growth without dead cells. A. SHH signaling agonist SAG enhances Panc1Fluc cell growth in a dose-dependent manner. Top: signal intensity analysis from bioluminescence image; Bottom: representative bioluminescence images; scale bar represents 1 cm. **represents *P*<0.01. B. SHH signaling agonist SAG enhances HT29Fluc cell growth in a dose-dependent manner. Top: signal intensity analysis from bioluminescence image; Bottom: representative bioluminescence images; scale bar represents 1 cm. *represents *P*<0.05, **represents *P*<0.01. C. Panc1Fluc cells treated with N-terminal fragment Shh demonstrate increased growth. Top: signal intensity analysis from bioluminescence image; Bottom: representative bioluminescence images; scale bar represents 1 cm. *represents *P*<0.05. D. HT29Fluc cells treated with N-terminal fragment Shh demonstrate increased growth. Top: signal intensity analysis from bioluminescence image; Bottom: representative bioluminescence images; scale bar represents 1 cm. **represents *P*<0.01.

Since Shh and Gli1 expression increased in irradiated Panc1 and HT29 cells and SHH signaling agonists enhanced dying tumor cell stimulated living tumor cell growth, we assumed that enhanced reporter cell growth was caused by SHH signal released from dying cells, thereby activating the SHH signaling pathway in living reporter cells should also cause cell growth. In order to verify our hypothesis we added the SHH signaling agonists SAG and the recombinant N-terminal fragment of Shh to wells containing Panc1 or HT29 reporter alone. Both SAG and the active form of Shh significantly increased Panc1 or HT29 reporter cell growth (Fig. 6). These findings suggest that the SHH signal released from dying cells resulted in reporter cell growth due to the activation of the SHH pathway in the reporter cells.

## Discussion

Tumor cell repopulation is a key process causing tumor relapse during cancer chemotherapy or radiotherapy [13]. Repopulation of surviving tumor cells can occur between dose fractions of either radiation or chemotherapy and can lead to treatment failure [14]. Developing strategies for the suppression of tumor cell repopulation is therefore crucial to the improvement of current radiotherapy or chemotherapy. However, there is limited understanding of the underlying biological mechanisms causing tumor repopulation. Huang et al. revealed that dying cancer cells could stimulate surviving cancer cell repopulation by caspase 3 mediated protein cleavage and consequent activation of growth promoting signals such as calcium-independent phospholipases A2 (iPLA2) [10]. In an effort to further elucidate this living tumor cell growth mechanism, our experiments sought to create an *in vitro* model of tumor repopulation in which dying cells treated with radiation signal living cells that survived the radiation to proliferate.

In this study, we further explored the concept of dying cells signaling surviving tumor cells to grow by investigating the role of the SHH signal pathway during this process. We found that SHH signaling could be activated by radiation. The irradiated tumor cells with higher Shh and Gli1 expression were associated with stronger tumor cell repopulation. Moreover, the dying cell stimulated living tumor cell growth could be further enhanced by SHH signaling agonists or recombinant N-terminal fragment of Shh and inhibited by SHH signaling antagonists or knockdown by Gli1shRNA. To our knowledge, this is the first study that showed SHH signaling activation in dying tumor cells playing an important role in the promotion of living tumor cell proliferation. We propose that this can serve as a model for tumor repopulation when some cells in a tumor are killed by radiation and the surviving, untreated cells are signaled to proliferate and cause tumor recurrence.

The idea of the SHH pathway contributing to tumor cell growth after radiation therapy is consistent with our current understanding of this pathway in tumor biology. The SHH signaling pathway is not only implicated in normal organ development and homeostasis, stem cell maintenance and proliferation [3], [4], but also in repair of normal tissue injury and tumor development [15], [16]. Glis in the SHH signaling pathway can directly bind to target genes and transcriptionally activate or repress these genes. In addition, SHH expression is positively correlated with EGFR expression. The blockade of the SHH signaling pathway enhances the anti-proliferative effect of the EGFR inhibitor through the down-regulation of EGFR expression [17]. Furthermore, SHH pathway is highly activated in pancreatic cancer stem cells and plays an important role in maintaining stemness [18]. It has been reported that combining gemcitabine with a hedgehog inhibitor eradicates cancer stem cells and results in reduced tumor growth [19]. Inhibition of SHH signaling also prolongs survival time of mice genetically pre-disposed to pancreatic cancer [20]. In essence, there is an abundance of current literature suggesting a role for SHH in tumor cell growth, and our experiments support that SHH signaling is important in the pathway of dying cell stimulated tumor growth.

In addition to playing a role in tumor development, the SHH signaling pathway has also been implicated in the cellular response to radiation in previous studies. Ptch1 heterozygotes, which is a transmembrane receptor of Shh ligand as repressor of SHH signaling, are hypersensitive to ionizing radiation induced tumorigenesis and may develop tumors such as basal cell carcinoma [21]. However, how and why radiation can induce the SHH pathway activation remains unclear.

Our study showed a differential effect of the SHH signaling antagonist cyclopamine in our two different cell lines. Specifically, the SHH signaling antagonist cyclopamine showed significant inhibition of HT29 cell growth but no effect on Panc1 cells growth. Most likely, these drugs have different physical interactions with Smo that can cause differences in cell-line sensitivity. Panc1 cells may not be susceptible to cyclopamine treatment, as reported previously [5], [22]. Possible explanations include differential ciliary transport of the drug that is needed to interact with Smo in different cell lines [23], slightly different physical drug interactions with Smo based on the cell-line specific mutations [24], or that resistance to Smo antagonists may arise from subversion of the pathway by cross talk from the RAS/Raf/MEK pathway [25].

In summary, based on the existing literature on the role of SHH signaling in tumor growth and the radiation response and our findings in this study, we believe that the SHH signaling plays an important role in tumor growth and relapse during radiotherapy or chemotherapy. The clinical implications of this study include a possible role for SHH inhibitors to enhance the efficacy and reduce the relapse due to radiation therapy. Our results also highlight the potential value of small molecular compounds or peptides blocking the SHH pathway as adjuvant during radiotherapy or chemotherapy. Essentially, the discovery of our proposed SHH signaling induced tumor cell repopulation has relevant clinical applications for future cancer treatment with radiation.
